# Supplementary material for: The interactome of intact mitochondria by cross-linking mass spectrometry provides evidence for coexisting respiratory supercomplexes
Source: Mol Cell Proteomics. 2017 Dec 8;17(2):216–32. doi: 10.1074/mcp.RA117.000470 (PMC5795388; doi:10.1074/mcp.RA117.000470)
Supplement: Supplemental Data [file supp_RA117.000470_133922_0_supp_23978_fzffwf.zip › spectra_annotation/mito_DR_spectra_annotation/245-1-14-1-2-1.pdf]

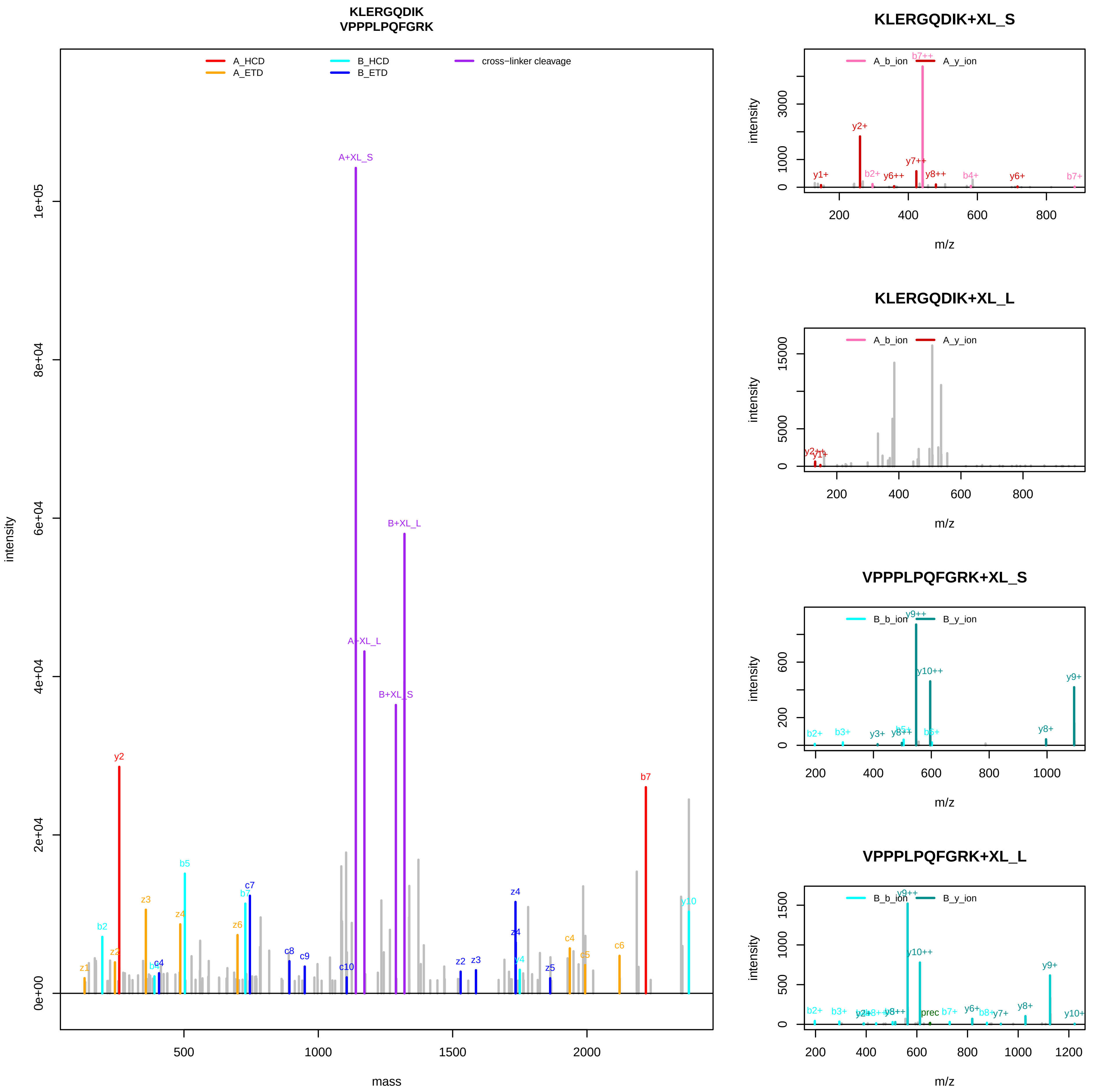
KLERGQDIK
VPPPLPQFGRK
A_HCD
A_ETD
B_HCD
B_ETD
cross−linker cleavage
A+XL_S
B+XL_L
A+XL_L
B+XL_S
y2
b7
b2
z1
z2
z3
z4
b4
c4
b5
z6
b7
c7
c8
c9
c10
z2
z3
z4
y4
z5
c4
c5
c6
y10
intensity
mass
0e+00
2e+04
4e+04
6e+04
8e+04
1e+05
500
1000
1500
2000
KLERGQDIK+XL_S
A_b_ion
A_y_ion
y1+
y2+
b2+
y6++
y7++
b7++
y8++
b4+
y6+
b7+
KLERGQDIK+XL_L
VPPPLPQFGRK+XL_S
B_b_ion
B_y_ion
y9++
y10++
y9+
y8+
b2+
b3+
y3+
b5+
b6+
VPPPLPQFGRK+XL_L
prec
b7+
y6+
b8+
y7+
y8+
y10+
m/z
